# Mechanical Suppression of Sonic Hedgehog Signaling by Nucleus Pulposus Cells Underlies Early Disc Degeneration in Mouse

**DOI:** 10.3390/cells15171506

**Published:** 2026-08-22

**Authors:** Sohrab Virk, Veeraj Shah, Vikrant Piprode, Claire Marie Kemp, Harshith Alluri, Kathleen F. Vincent, Ravi Krishnan, Justin Hong, Todd J. Albert, Chitra L. Dahia

**Affiliations:** 1 Spine Service, Hospital for Special Surgery, New York, NY 10021, USA; 2 Orthopedic Soft Tissue Research Program, Hospital for Special Surgery, New York, NY 10021, USA; 3 Department of Cell and Developmental Biology, Weill Cornell Medical College, New York, NY 10021, USA; 4 Weill Cornell Medical College, New York, NY 10021, USA

**Keywords:** sonic hedgehog, nucleus pulposus, static compression, immobility, tail-loop model, disc degeneration, *Piezo1*, mechanotransduction

## Abstract

Sonic hedgehog (SHH) expression by nucleus pulposus (NP) cells is important for intervertebral disc development and maintenance. The lumbosacral disc, the most immobile region of the spine, lies adjacent to the sacrum and is most vulnerable to degeneration. We hypothesized that a lack of mobility represses SHH expression by NP cells, leading to degenerative changes in the intervertebral disc. To test this hypothesis, we employed a *Shh*-LacZ reporter mouse and a tail-loop surgical model of constant compression and immobility. Using a comprehensive level-by-level analysis, we first determined the coccygeal level most affected by geometric and histopathological changes. We next determined the effects of constant compression and immobility on the subset of SHH-expressing cells compared with sham controls. Multiplex qPCR analysis validated a decline in *Shh* and its target *Gli1* expression by NP cells in the tail-looped discs that was associated with an increase in *Piezo1* expression compared to sham controls. In summary, the findings support a model in which restricted mobility and sustained compression in lumbosacral discs accelerate pathology by prematurely silencing developmental signaling programs, such as SHH, required for NP homeostasis.

## 1. Introduction

Degeneration of the intervertebral disc (IVD) is a common cause of chronic low back pain, the greatest contributor to global disability [1,2]. IVDs function as load-bearing joints that connect adjacent vertebral bodies while permitting spinal flexibility. Each disc consists of a gelatinous nucleus pulposus (NP) surrounded by the fibrous annulus fibrosus (AF) and bounded by cartilaginous endplates (EP). Degeneration disrupts this coordinated structure, leading to impaired load distribution, altered biomechanics, and progressive tissue failure, resulting in chronic pain. Although IVD degeneration can occur throughout the spine, it disproportionately affects the lumbosacral level, a biomechanically unique transition zone subjected to sustained load and restricted mobility [3,4,5]. Like humans, a high prevalence of IVD degeneration at the lumbosacral level is reported in mice [4,6,7,8,9], the most widely used pre-clinical model of IVD biology. Despite the clinical importance of this region, the biological mechanisms that render lumbosacral IVDs particularly vulnerable to early degeneration remain poorly understood.

Anatomically, the lumbosacral IVD (L5/S1 in humans and L6/S1 in mice) is positioned immediately superior to the sacrum, which is immobilized between the hip joints. Consequently, lumbosacral discs have a reduced range of motion, are stiffer, and experience higher compressive loads compared to the more cranial lumbar IVDs [10,11]. Similar mechanical conditions characterize the sacral IVDs, which are the least mobile segments of the spine that fuse by skeletal maturity. These observations suggest that sustained compression and a limited range of motion may be key drivers of regional disc vulnerability; however, the molecular pathways linking mechanical loading to accelerated degeneration at this level remain unclear.

NP cells are central regulators of IVD homeostasis and are descendants of the embryonic notochord. Remarkably, postnatal NP cells retain expression of sonic hedgehog (SHH), a developmental morphogen essential for disc growth, maintenance, and signaling to the surrounding AF and EP tissues of the IVD [12,13,14,15]. A decline in SHH signaling is a defining feature of disc aging and degeneration and is associated with the terminal differentiation of NP cells into a chondrocyte-like phenotype [9,12,14,15]. Importantly, the loss of SHH-expressing NP cells occurs earlier and more dramatically in lumbosacral IVDs than in adjacent lumbar levels and correlates with disc pathology [9]. In the sacrum, complete loss of SHH expression coincides with disc fusion, and genetically activating hedgehog signaling in NP cells is sufficient to revert their differentiation and sacral fusion, establishing a causal relationship between SHH signaling and disc fate [15]. These findings suggest that reduced mobility and chronic compression may actively suppress SHH expression in NP cells, thereby accelerating degeneration in mechanically constrained IVDs.

Mechanical loading is an important regulator of IVD structure and biology [16,17,18,19,20,21,22,23,24]. However, in vivo and ex vivo studies across species demonstrate that chronic static compression promotes matrix breakdown, cellular dysfunction, and disc degeneration [25,26,27,28,29,30,31,32,33,34,35,36,37,38]. The rodent tail-loop model imposes sustained compression and immobility across multiple IVD levels and introduces asymmetric, wedged loading that more closely resembles the lumbar disc geometry [26,27,39]. However, whether static compression and immobility directly alter developmental signaling pathways, such as SHH signaling in NP cells, has not been established in vivo.

This study aimed to determine whether limitations in range of motion, along with chronic static compression, directly suppress SHH expression as an early response in NP cells in vivo and thereby accelerate IVD degeneration. Using a *Shh*-LacZ reporter mouse allele [40] combined with a multi-level tail-loop compression paradigm, we systematically assessed how sustained mechanical constraint alters disc geometry, *Shh* expression, and matrix organization. We also evaluate the impact of tail-loop immobilization on the IVD structure and geometry in both the sagittal and coronal planes. In addition, we interrogate the mechanobiological relationship between altered loading and the loss of key developmental signaling within the IVD.

The significance of this work lies in identifying the mechanical repression of SHH expression as a mechanistic link between chronic compression and region-specific disc vulnerability. These findings provide a biological explanation for the increased susceptibility of lumbosacral discs—where mobility is restricted, and loading is sustained—to early degeneration. More broadly, this study establishes developmental signaling pathways as active mechanosensitive regulators in the adult IVD, offering new insight into how biomechanical environments initiate degenerative cascades and highlighting SHH signaling as a potential target for early therapeutic intervention.

## 2. Materials and Methods

### 2.1. Animals

Skeletally mature male (*n* = 16) and female (*n* = 12) *Shh*-LacZ reporter mice (*Shh-nLz*; *Shh^tm1.1Ahk/J^*; JAX #035066, [40]) on an FVB background were maintained under a 12 h light/dark cycle with food and water ad libitum. All procedures adhered to the NIH Guide for the Care and Use of Laboratory Animals and were approved by the Institutional Animal Care and Use Committee (IACUC), protocol number 2016-0026, 7 February 2019. Genotyping was performed shortly after birth using DNA from toe clip biopsies, allele-specific primers, and standard PCR followed by agarose gel electrophoresis.

### 2.2. Tail-Loop Immobilization Surgery

Tail immobilization was performed as previously described [27] on 12-week-old *Shh*-LacZ reporter mice (tail-looped: *n* = 18, 8 females; sham controls: *n* = 12, 5 females). Mice received meloxicam (2 mg/kg, s.c.) pre-operatively for analgesia. Pre-operative radiographs were used to identify coccygeal vertebrae Co5 and Co13. A sterile 4-0 surgical steel monofilament loop was passed through the skin to secure Co5 to Co13, after which vertebrae distal to Co13 were amputated under aseptic conditions. This configuration immobilized IVDs between Co5 and Co13 to generate sustained compressive loading. Bupivacaine (0.25%) was applied locally post-procedure. Mice were euthanized 4–8 weeks post-surgery, and Co5–Co13 coccygeal segments were harvested for analysis.

For downstream analyses, cohorts were subdivided as follows: (1) sagittal sectioning of Co5–Co13 levels; (2) microdissection of NP cells from Co5–Co13 for qPCR analysis; and (3) coronal sectioning of Co8–Co10 for X-gal and histochemical staining with NP microdissection from Co5–Co8 and Co10–Co13 for qPCR analysis.

### 2.3. Radiographic Imaging and Quantification

Radiographs (UltraFocus, Faxitron Bioptics, Tucson, AZ, USA) were obtained pre-operatively, immediately post-looping, and one-month post-surgery from both the tail-looped (*n* = 9) and sham groups (*n* = 3). Radiograph images were analyzed using the Nikon NIS-Elements Advanced Research software (Nikon, Tokyo, Japan, Vs. 6.20). The IVD wedging ratio (B/A) was calculated as the tensioned disc height (B) divided by the compressed disc height (A). The disc angle was calculated using the “angle tool” in NIS-Elements. For unwedged discs, the intervertebral angles between adjacent vertebral bodies were measured using the “angle between lines” method, whereas, for wedged discs, the standard “angle tool” was used. Changes in disc wedging and disc angle were assessed using non-parametric Wilcoxon matched-pairs signed-rank tests with Holm–Šídák correction for multiple comparisons, comparing subjects before and after surgery within each cohort (sham control and tail-loop). Adjusted *p*-values are reported. The charts present the Gaussian curve using non-linear regression across levels within a cohort.

### 2.4. Tissue Processing, Histochemical Staining, and Imaging

Tail segments were fixed in buffered 0.4% paraformaldehyde (PFA; Sigma-Aldrich, Saint Louis, MO, USA, P6148-500G) and 0.2% glutaraldehyde (Fisher Scientific, Waltham, MA, USA, 50-262-08) for 6 h at 4 °C with gentle agitation, rinsed in cold PBS (1×; Laboratory Disposable Products, Medford, NJ, USA, 46-013-CM), and decalcified in 0.5 M EDTA (pH 7.4; Sigma-Aldrich, E9884) at 4 °C for 2 days (coronal sections) to 21 days (sagittal sections). Samples were embedded in Tissue-Tek^®^ O.C.T. compound (Fisher Scientific, 14-373-65) and cryosectioned at 8 µm using a Leica CM1950 cryostat (Wetzlar, Germany). Slides were stored at −80 °C until further use.

Hematoxylin and eosin (H&E) staining was performed using a standard protocol (hematoxylin: Sigma, Burlington, MA, USA, GHS1128-4L; eosin: Sigma, Burlington, MA, USA, HT110380-2.5L) to analyze structural changes. Safranin-O/Fast Green staining using 0.1% Safranin-O (Sigma, Burlington, MA, USA, 50240) and 0.001% Fast Green (Sigma, Burlington, MA, USA, 42053) was performed for 20 min to assess proteoglycans and collagen. Picrosirius Red staining using 0.1% Sirius Red (Polysciences 09400) in saturated picric acid (Sigma, Burlington, MA, USA, P6744) was conducted for 30 min to evaluate collagen birefringence. Movat’s Pentachrome staining was performed using the Modified Russell–Movat kit (Abcam, Cambridge, UK, ab245884), according to the manufacturer’s instructions. Sections were dehydrated in an ethanol gradient, cleared in xylene, and mounted in xylene-based media (Fisherbrand, Pittsburgh, PA, USA, 122442). Brightfield images of histochemically stained slides were captured on a Nikon Eclipse Ei microscope with a DS-Fi2 camera and the NIS-Elements AR software (Vs. 6.20). Standard illumination was used for the imaging of H&E- and Safranin-O/Fast Green-stained sections. X-gal sections were imaged in brightfield and with differential interference contrast (DIC) to visualize the disc structure and cell morphology. Picrosirius Red sections were imaged using crossed polarizers to assess collagen organization.

### 2.5. Morphometric Analyses

Structural changes were quantified using H&E-stained mid-sagittal and mid-coronal sections. Changes in NP and AF geometry were quantified by measuring changes in aspect ratio and area in a given region using the NIS-Elements Advanced Research software. The aspect ratio was calculated using the formula “aspect ratio = width/height”. The NP width and height were each measured at three locations per serial section; values were averaged across three serial sections to yield one value per IVD. The NP area was obtained by manual ROI tracing along the NP boundary across three serial sections and averaged per IVD. The AF region on either side was analyzed separately. To calculate the AF aspect ratio, three measurements were obtained at each disc height and width in each serial section, and the values were averaged across the three serial sections. The AF area was quantified by manual ROI tracing on each side and averaged across sections. All NP parameters in the sagittal plane were analyzed using two-way ANOVA with Šidák’s multiple-comparison test. All AF parameters in sagittal were analyzed using a mixed-effects model with Tukey’s multiple-comparison test to compare the controls and the compressed and tensioned sides of the looped discs. NP and AF parameters in coronal sections were analyzed using an unpaired Welch’s *t*-test.

### 2.6. Histopathological Scoring

Histopathological analysis was performed on de-identified H&E-stained sagittal and coronal sections from tail-looped and control mice using the Melgoza and Chenna mouse IVD scoring system [41]. Four regions—NP, AF, EP, and interface—were assessed using region-specific features. NP and AF were scored 0–3; EP and interface were scored 0–2. Scores from two independent raters were averaged per region to yield a final regional score per IVD. Region-specific histopathological scores for the NP, AF, EP, and interface regions of the disc from each cohort were statistically analyzed using two-way ANOVA with Šidák’s multiple-comparison tests. The scores for each region were summed to generate cumulative histopathological scores per disc, which were analyzed using a mixed-effects model with Šidák’s multiple-comparison tests. Changes in NP phenotype were assessed by manually counting and categorizing NP cells in each IVD as reticular-NP, small-NP, or chondrocyte-like cell-NP (CLC-NP). Quantification was performed using three serial sections per biological sample, and the resulting averages were analyzed with a mixed-effects model and Šidák’s multiple-comparison tests to compare the tail-loop group to the sham controls.

### 2.7. Immunofluorescence Staining

Immunostaining was conducted as previously described [9,13,15]. Following permeabilization with chilled methanol at −20 °C, sections were blocked in blocking buffer for 1 h. Sections were then incubated overnight at 4 °C in a humidified chamber with KRT19/CK19 primary antibody (1:50; TROMA-III; Developmental Studies Hybridoma Bank, Iowa City, IA, USA). The following day, sections were washed in PBS and incubated with Alexa Fluor 647-conjugated secondary antibody (Jackson ImmunoResearch, West Grove, PA, USA) diluted 1:200 for 1 h. Nuclei were counterstained with DAPI (1:5000 in PBS; D1306; Life Technologies, Carlsbad, CA, USA) during the final PBS wash. Slides were mounted using Prolong^TM^ Gold mounting media (P36934; Life Technologies). Images were acquired using a Zyla sCMOS camera on a Nikon Eclipse epifluorescence microscope (Tokyo, Japan) with the NIS-Elements Advanced Research software. IVD compartments were delineated using a DIC filter to define the NP boundary. Results were analyzed using an unpaired two-tailed Welch’s *t*-test to compare tail-looped and sham cohorts.

### 2.8. TUNEL Assay for Apoptosis Detection

Cell death was evaluated using the Click-iT™ Plus TUNEL Assay for In Situ Apoptosis Detection with Alexa Fluor™ 594 dye (Thermo Fisher Scientific, Waltham, MA, USA, Cat. No. C10618). Slides were permeabilized with Proteinase K and equilibrated in 1× TdT reaction buffer; they were then labeled with terminal deoxynucleotidyl transferase and EdUTP for 60 min at 37 °C. Sections were subsequently incubated with the Click-iT™ Plus TUNEL reaction cocktail containing Alexa Fluor™ picolyl azide for 30 min at 37 °C, protected from light. Nuclei were counterstained with DAPI, slides were mounted in ProLong™ Gold, and images were acquired as previously described. Cell death in NP cells was quantified by determining the proportion of DAPI-positive NP cells that were also TUNEL-positive. Statistical analysis was performed using an unpaired two-tailed Welch’s *t*-test to compare tail-looped and sham cohorts.

### 2.9. Cell Number Quantification

Slides were washed, and nuclei were stained with DAPI. Slides were mounted in ProLong™ Gold, and images were acquired as described above. DAPI-stained nuclei were quantified in the NP and AF compartments with the automated counting module in NIS-Elements and normalized to the tissue area (cells/mm^2^). Three serial sections per IVD were analyzed and averaged per biological replicate. The number of NP cells in the sagittal plane was analyzed using two-way ANOVA and Šídák’s multiple-comparison test. The number of AF cells in the sagittal plane, comparing the compressed and tensioned sides of the looped and control groups, was analyzed using a mixed-effects model with Tukey’s multiple-comparison test. The NP cell count per disc in the coronal plane was analyzed using an unpaired Welch’s *t*-test. The AF cell count per disc in the coronal plane was obtained by averaging both sides per disc in each cohort and analyzed using an unpaired Welch’s *t*-test.

### 2.10. X-Gal Staining and Quantification (Shh-LacZ)

Coronal sections from Co8–Co10 IVDs (tail-looped *n* = 5; controls *n* = 5) were fixed in 0.4% PFA (Sigma, Burlington, MA, USA, P6148-500G) and 0.2% glutaraldehyde (Sigma, Burlington, MA, USA, G7651) for 2 min, rinsed in 2 mM MgCl_2_ (Sigma, Burlington, MA, USA, M8266), and permeabilized for 20 min in pre-incubation buffer (2 mM MgCl_2_, 0.01% sodium deoxycholate [Sigma, Burlington, MA, USA, D6750], 0.02% NP-40/IGEPAL CA-630 [Sigma, Burlington, MA, USA, 18896] in PBS). Sections were incubated at 37 °C for 4 h in freshly prepared X-gal solution (2 mM MgCl_2_, 0.01% sodium deoxycholate, 0.02% NP-40, 5 mM potassium ferricyanide [Sigma, Burlington, MA, USA, 244023], 5 mM potassium ferrocyanide [Sigma, Burlington, MA, USA, P3289], and 1 mg/mL X-gal from a 20 mg/mL stock [Invitrogen, Waltham, MA, USA, B1690] in PBS) in a humidified, light-protected chamber. After washing in 2 mM MgCl_2_, sections were counterstained with Nuclear Fast Red (Sigma, Burlington, MA, USA, N3020) and post-fixed in 4% PFA for 2 min; they were then dehydrated, cleared, and mounted (see above).

Images were acquired with a DS-Fi2 camera on a Nikon microscope (Tokyo, Japan) using the NIS-Elements Advanced Research software. The ROI was defined around the NP region to quantify LacZ+ (blue) *Shh*-expressing relative to the total NP cell number. Over three serial sections were quantified per biological replicate, and averages were used to calculate the fraction of *Shh*-expressing LacZ+ NP cells. Results were analyzed using an unpaired two-tailed Welch’s *t*-test comparing the tail-looped vs. sham cohorts.

### 2.11. RNA Isolation and Quantitative PCR

NP cells were microdissected from Co5–Co13 of the tail-looped individuals (*n* = 6) and sham controls (*n* = 3) and collected and processed for RNA isolation and multiplex qPCR analysis. The experiment was repeated using NP cells collected from Co5–Co8 and Co10–Co13 in tail-looped (*n* = 5) and sham control (*n* = 3) littermates, where Co8–Co10 were used for X-gal staining and histological evaluation in the coronal plane. Briefly, total RNA was extracted using Tri-Reagent (Sigma, Burlington, MA, USA, 93289) and purified on RNeasy Mini columns (Qiagen, Germantown, MD, USA, 74104). RNA integrity was assessed using BioAnalyzer (Santa Clara, CA, USA, Agilent 2100) and samples with RIN > 8 were reverse-transcribed using SuperScript™ IV VILO (Invitrogen, Waltham, MA, USA, 11756050). Multiplex qPCR was performed on a Bio-Rad CFX96 (Hercules, CA, USA) using TaqMan assays for *Shh* (FAM-MGB; Thermo Fisher, Waltham, MA, USA, Mm00436528_m1), *Gli1* (FAM-MGB; Thermo Fisher, Waltham, MA, USA, Mm00494654_m1), *Piezo1* (FAM-MGB; Thermo Fisher, Waltham, MA, USA, Mm01241549_m1), and *Cox2* (FAM-MGB; Thermo Fisher, Waltham, MA, USA, Mm03294838_g1), with primer-limited *Gapdh* (VIC-MGB; Thermo Fisher, Waltham, MA, USA, Mm99999915_g1) as the internal control. ΔCT values were calculated relative to *B2m*, and log_2_ fold changes were derived. Results were analyzed using an unpaired two-tailed Welch’s *t*-test comparing the tail-looped vs. sham cohorts.

### 2.12. Statistical Analysis

All statistical analyses were conducted in GraphPad Prism v10. Unless otherwise stated, *p* < 0.05 and adjusted *p* < 0.05 were considered statistically significant. Details for each analysis are specified in the respective section and the figure legends.

## 3. Results

### 3.1. Tail-Loop Immobilization Alters Disc Geometry

**Figure 1:**
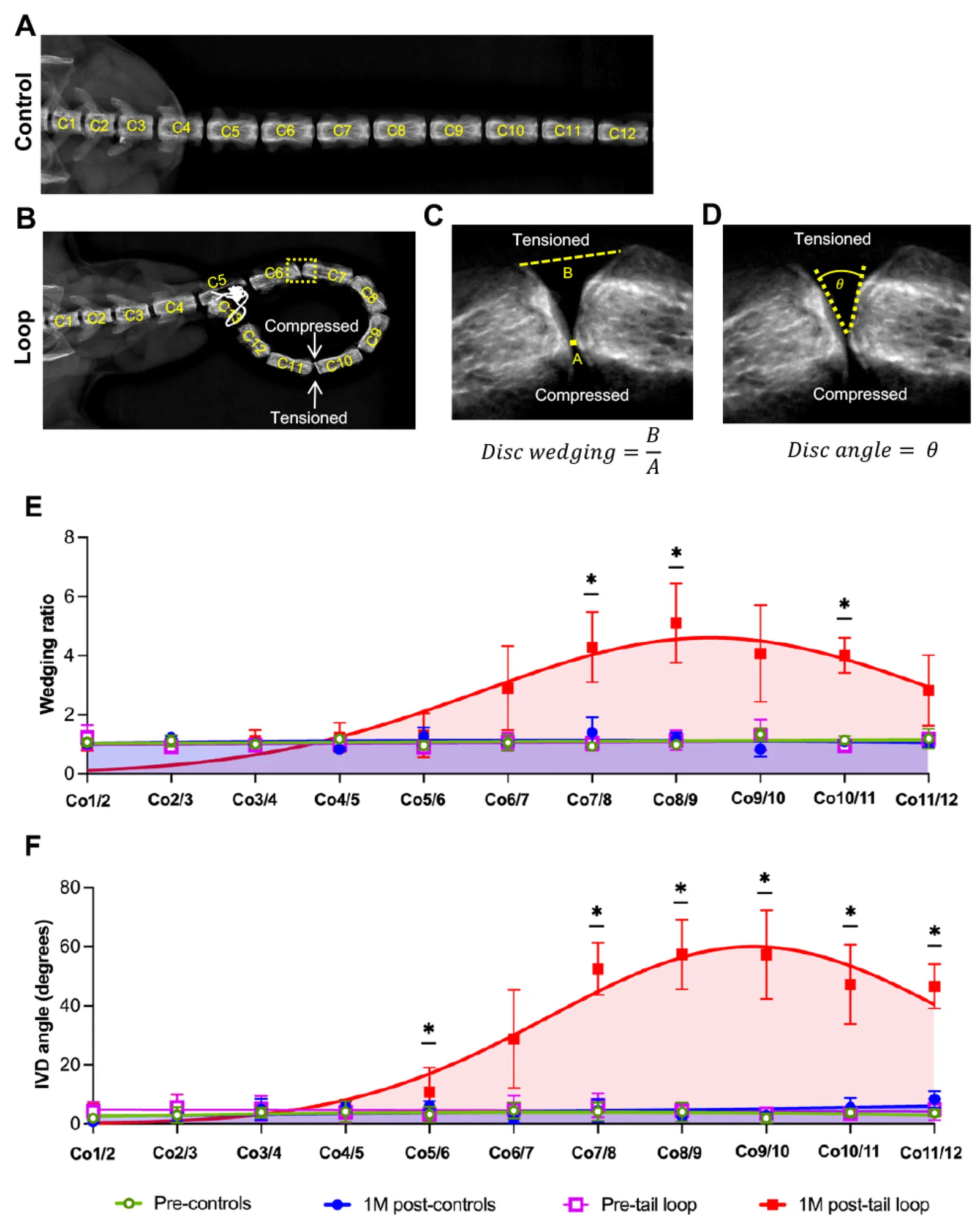
Tail-loop immobilization induces IVD wedging. (**A**,**B**) Representative dorsal X-ray images of the mouse tail, showing vertebral levels (Co1–Co13) in control (**A**) and tail-loop-immobilized (looped) (**B**) cohorts. The tail-loop immobilization model fixes vertebrae from Co5 to Co13, creating an “compressed” (inside) and “tensioned” (outside) region. (**C**,**D**) Schematic representation of quantitative radiographic parameters. The disc wedging ratio was calculated as B/A, where A and B represent the compressed- and tensioned-side disc heights, respectively (**C**). The disc angle (θ) was defined as the angle formed between adjacent vertebral endplates (**D**). (**E**) Quantification of disc wedging ratio across vertebral levels (Co1–Co12) in pre- and post-surgical cohorts. (**F**) Quantification of IVD angle deformation across vertebral levels in the same groups. Statistical analyses were performed using the non-parametric Wilcoxon matched-pairs signed-rank test with Holm–Šídák correction for multiple comparisons. The data are presented to show the Gaussian curve using non-linear regression across levels within a cohort. In (**E**,**F**), the pink and blue filling shows the area under the curve for the 1-month post-loop and post-control cases, respectively. Error bars represent mean ± SD. Asterisks denote statistical significance, calculated using a multiple-comparison test. * adj*p* < 0.05.

Lumbosacral IVDs in humans and mice are physiological wedges, with the posterior (human) or dorsal (mouse) side compressed relative to their anterior and ventral sides. To determine how sustained static loading alters the IVD geometry, we first analyzed coccygeal disc alignment and morphology following surgical tail-loop immobilization. In sham controls, coccygeal vertebrae from Co1 to Co12 remained uniformly aligned along the tail axis (Figure 1A), with no detectable post-surgical changes. In contrast, tail-loop immobilization produced a stable curvature spanning Co5 to Co13, generating distinct regions under compressive and tensile (stretched) forces (Figure 1B).

To quantify geometric changes, we measured disc wedging (tensioned/compressed ratio; Figure 1C) and the disc angle between adjacent vertebrae (θ; Figure 1D). The wedging ratios in sham controls were unchanged between pre- and post-surgery time points (Figure 1E). Tail-loop immobilization resulted in a significant and progressive increase in disc wedging toward the center of the loop at one month post-surgery compared to both pre-surgery values (adj*p* < 0.05). Following tail-loop immobilization, the disc wedging ratio increased from Co5 to Co8 levels. A decline in disc wedging was observed from Co8 to Co12 (Figure 1E).

The disc angle measurements mirrored the findings from the disc wedging analysis. No significant changes in disc angle were observed in controls post-sham surgery (Figure 1F). In contrast, tail-looped mice exhibited a bell-shaped Gaussian curve for the disc angle that increased from Co5 to Co8–Co10, which represents the middle of the loop, followed by a decline at the end of the loop. The changes in disc angle at one month post-looping were significant compared with pre-surgical measurements (adj*p* < 0.05; Figure 1F). Together, these data identify Co8–Co11, which are midway in the loop, as the geometrically most affected disc levels following chronic compressive loading and immobilization, and they were selected for subsequent analyses.

### 3.2. Structural Changes Mirror Disc Geometry Following Tail-Loop Immobilization

**Figure 2:**
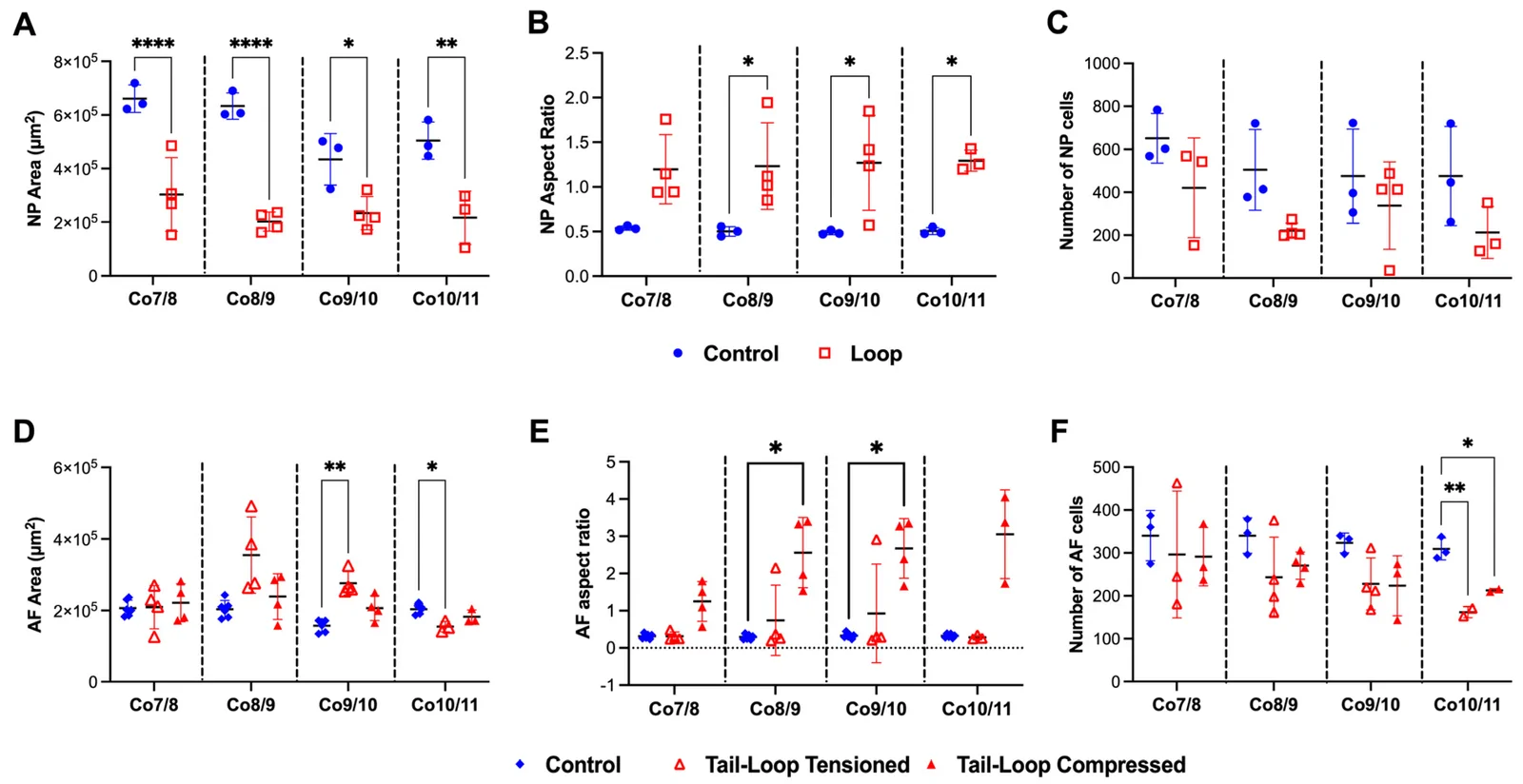
Tail-loop immobilization induces structural remodeling of IVD compartments. Quantification of histomorphometry parameters across Co7/8 to Co10/11 coccygeal levels was performed using H&E-stained images and included NP area (**A**), NP width-to-height aspect ratio (**B**), NP cell number (**C**), AF area (**D**), AF width-to-height aspect ratio (**E**), and AF cell number (**F**) between sham control (*n* = 3) and tail-looped (*n* = 4) cohorts. Statistical analysis of NP area, width-to-height aspect ratio, and cell number was conducted using two-way ANOVA with Šidák’s multiple-comparison test. Statistical analysis of AF area, aspect ratio, and cell count was performed using a mixed-effects model with Tukey’s multiple-comparison test. Data points represent individual discs. Error bars indicate mean ± SD. Asterisks denote statistical significance. * *p* < 0.05, ** *p* < 0.01, **** *p* < 0.0001.

Next, we assessed structural changes in the NP and AF at Co8–Co11 at the microscopic level using H&E-stained sagittal sections from sham controls and tail-looped mice at four to eight weeks following surgery. As no differences were observed between the two time points, the data were merged for analysis (Figure 2A–C, Supplemental Figure S1).

Quantitative histomorphometry revealed a marked reduction in NP area (*p* < 0.05, Figure 2A) across all analyzed levels and in the NP width-to-height aspect ratio (*p* < 0.05, Figure 2B) across the Co8 to Co11 levels in tail-looped discs relative to controls. In contrast, the NP cell number was unchanged, indicating that geometric deformation occurred without overt cell loss (Figure 2C, Supplemental Figure S2A). However, the NP appeared to adapt to the asymmetrical structure and was forced closer to the tensioned side of the IVD.

The AF was measured and analyzed separately on the compressed and tensioned sides to resolve asymmetric responses to loading. The tensioned AF exhibited a significant increase in area at the Co9/10 level (*p* < 0.01) but a reduction at Co10/11 (*p* < 0.05) relative to controls (Figure 2D). Consistent with disc wedging, the AF width-to-height aspect ratio was significantly altered on the compressed side compared with both the tensioned side and controls at the Co8 to Co10 levels (*p* < 0.05). In contrast, no significant change was observed on the tensioned side of the looped discs relative to controls (Figure 2E). Furthermore, AF cellularity was significantly reduced on both the compressed and tensioned sides at the Co10/11 level (*p* < 0.05), while the AF cell numbers at adjacent levels remained unchanged (Figure 2F, Supplemental Figure S2A).

Collectively, these findings demonstrate that chronic tail-loop immobilization drives the pronounced, asymmetric structural remodeling of both the NP and AF compartments of the Co8–Co10 discs, which are in the middle of the looped segment, when analyzed in the sagittal plane. While the remaining discs in the looped segment continue to experience static compression and reduced mobility, they are only modestly affected at the structural level.

### 3.3. Structural Alteration Is Associated with Modest Degeneration Following Tail-Loop Immobilization

**Figure 3:**
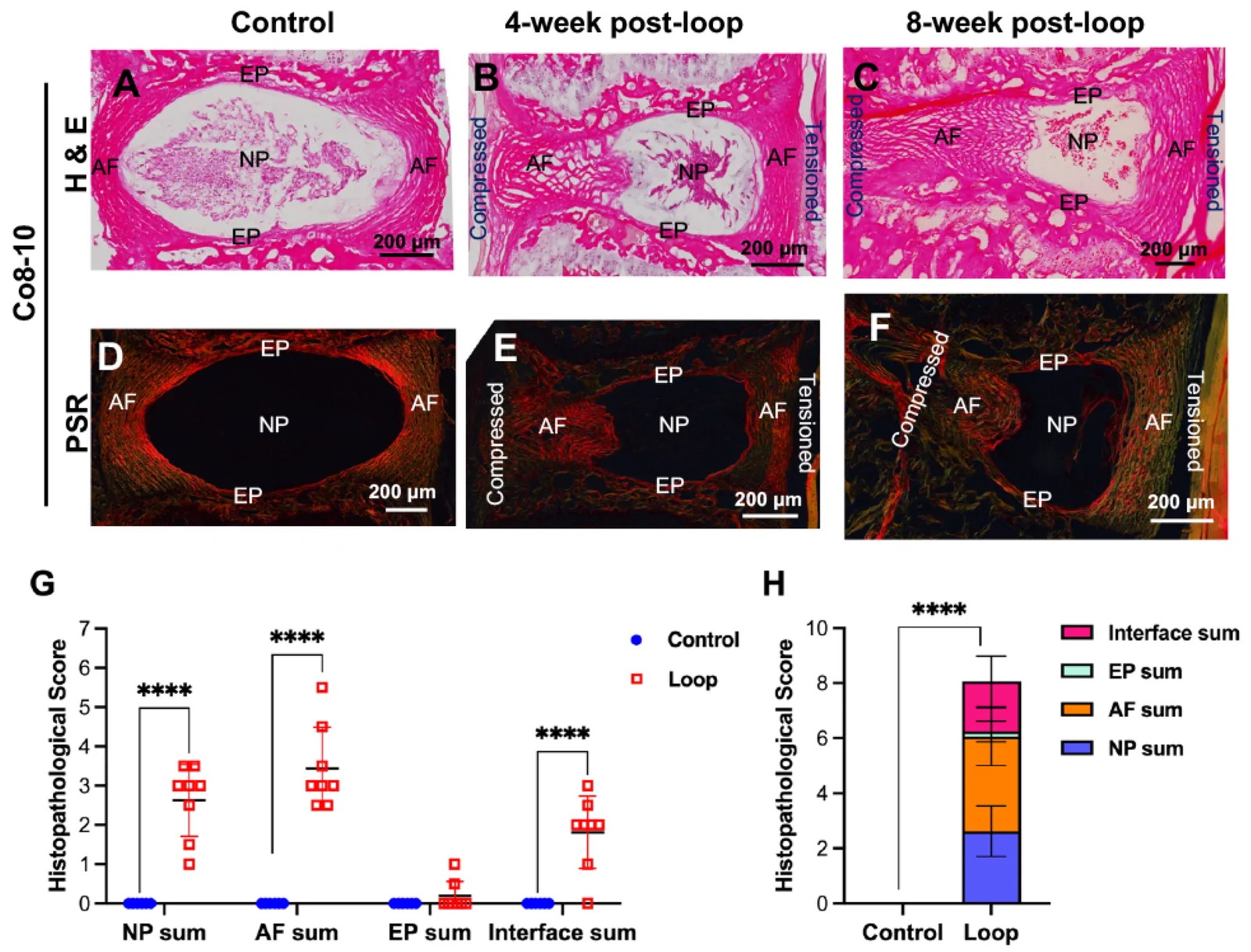
Chronic tail-loop immobilization drives IVD degeneration. (**A**–**C**) Representative H&E-stained sagittal sections of Co8–10 IVDs from sham control (**A**), 4-week (**B**), and 8-week (**C**) post-tail-loop immobilization cohorts. (**D**–**F**) Representative Picrosirius Red-stained sagittal serial sections imaged using cross-polars for collagen birefringence from Co8–10 IVDs from sham control (**D**), 4-week (**E**), and 8-week (**F**) post-tail-loop immobilization cohorts, visualized under dark-field microscopy. (**G**,**H**) Histopathological scoring was performed using H&E-stained sagittal sections from Co8 to Co10 IVDs of the control (*n* = 6 IVDs) and tail-looped (*n* = 8 IVDs, combining 4- and 8-week data) cohorts. (**G**) Sum of histopathological scores for each region of the coccygeal IVDs representing NP, AF, EP, and the interface regions, as analyzed using two-way ANOVA with Šidák’s multiple-comparison tests. (**H**) Cumulative histopathological scores obtained by adding the scores for each region, including NP, AF, EP, and the interface of control and tail-looped IVDs sectioned in the sagittal plane. Statistical significance was determined using a mixed-effects model and Šidák’s multiple-comparison tests. Data points represent individual discs. Error bars indicate mean ± SD. Asterisks denote statistical significance. **** *p* < 0.0001. NP, nucleus pulposus. AF, annulus fibrosus. EP, endplate.

Next, we analyzed the histopathological consequences of tail-loop immobilization at 4 and 8 weeks post-surgery, focusing on the Co8 to Co10 IVD levels, which were structurally most affected. In sham controls, IVDs exhibited a normal architecture characterized by a centrally located reticular spread-out NP and well-organized AF lamellae (Figure 3A). In contrast, looped discs displayed minor structural changes, including NP clumping and pronounced asymmetry between the compressed and tensioned sides of the mechanically wedged discs (Figure 3B,C). These structural changes were consistent between the four- and eight-week post-surgery timepoints.

Using Safranin O–Fast Green staining, previous reports showed subtle changes in proteoglycan and collagen matrix organization following tail-loop immobilization [27,39]. Hence, to gain further insight into the effects of chronic mechanical compression on the collagen quality of the AF, we utilized Picrosirius Red staining and polarized microscopy, which revealed well-aligned, uniformly red birefringent collagen fibrils in the AF of control discs, consistent with thick, organized collagen bundles (Figure 3D). Following tail-loop immobilization, collagen organization was deteriorated to a similar extent at 4 to 8 weeks post-surgery (Figure 3E,F). The tensioned AF exhibited increased green birefringence, indicating thinner, weaker collagen fibrils, whereas the compressed AF—particularly on the side adjacent to the NP—retained red birefringence but appeared disorganized and fragmented, consistent with altered load transmission (Figure 3E,F).

As no notable differences were observed between the 4- and 8-week post-looping time points, we combined the histopathological data from these time points for analysis. We analyzed the effects of static loading and immobilization relative to sham controls. Histopathological scoring was conducted using the Melgoza and Chenna mouse scoring system [41]. Histopathological scoring of each IVD region revealed a modest but significant increase in total degeneration scores in the compressed discs within the loop (*p* < 0.0001), driven primarily by AF scores, followed by NP, and the subtle disruption of the NP-AF interface (Figure 3G). In contrast, EP scores were unchanged (Figure 3G). AF degeneration scores were consistent with an increase in the tensioned-to-compressed AF ratio, indicating disruption of the inner annulus. NP scores were driven by clumping and minor fibrosis. An analysis of the cumulative score for the entire IVD demonstrated mild pathology in looped discs compared with controls (Figure 3H), consistent with previous reports [27,39]. Together, these data indicate that tail-loop immobilization produces level-dependent, modest asymmetric disc pathology characterized by NP deformation, collagen disorganization, and load-specific AF remodeling. Additionally, the Co8–Co10 IVD levels are most affected at both the geometrical and histological levels.

### 3.4. Coronal Analyses Reveal Subtle Matrix Alterations but Underestimate Altered Disc Geometry

Given the pronounced asymmetry observed at the Co8–Co10 levels in the sagittal plane, we next examined these levels in the coronal plane to assess whether similar structural changes were evident in the overall disc geometry post-looping, irrespective of the analysis orientation. Considering that the 4- and 8-week time points post-looping yielded similar findings in the sagittal plane, we analyzed earlier time points—between 4 and 6 weeks post-looping—to examine early molecular changes due to static compression in the coronal plane.

**Figure 4:**
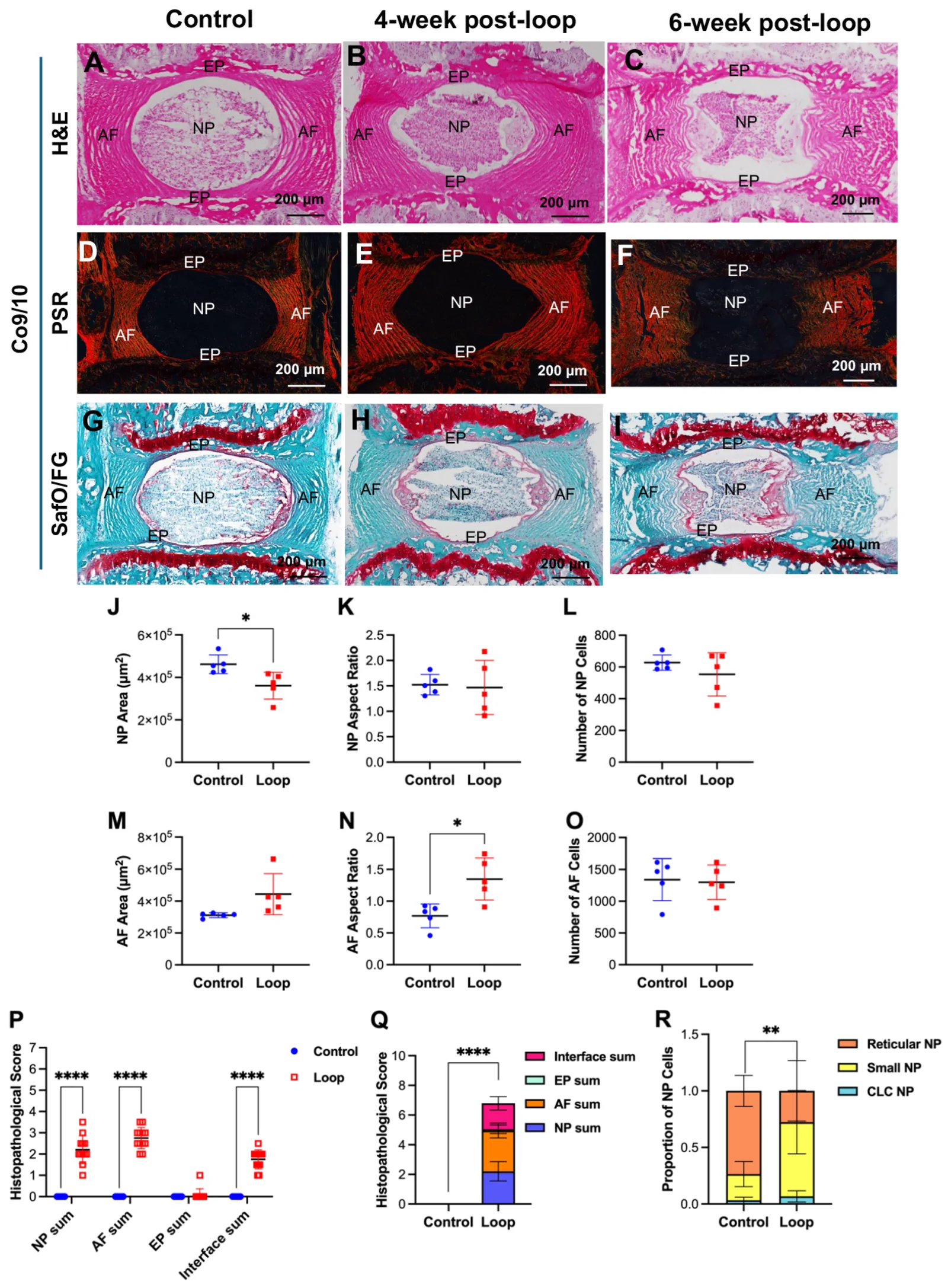
Coronal analyses reveal subtle alterations. (**A**–**C**) Representative H&E-stained mid-coronal sections of Co9/10 coccygeal IVDs from sham controls (**A**) and 4 weeks (**B**) and 6 weeks (**C**) post-tail-loop immobilization. (**D**–**F**) Representative Picrosirius Red-stained mid-coronal sections of Co9/10 coccygeal IVDs imaged using cross-polars and showing collagen fiber organization in the discs between cohorts. (**G**–**I**), Representative Safranin O–Fast Green-stained mid-coronal sections of Co9/10 coccygeal IVDs, illustrating proteoglycan and collagen distribution within IVD compartments between cohorts. (**J**–**O**) Statistical analysis of morphometric parameters in NP and AF regions of Co9/10 IVDs between control (*n* = 5) and loop (*n* = 5) cohorts, performed using unpaired Welch’s *t*-test. Morphometric parameters include NP area (**J**), NP width-to-height aspect ratio (**K**), NP cell number (**L**), AF area (**M**), AF width-to-height aspect ratio (**N**), and AF cell number (**O**). (**P**,**Q**) Histopathological scoring was performed using H&E-stained mid-coronal sections from the Co8 to Co10 coccygeal IVDs in the control (*n* = 6) and tail-looped (*n* = 12, combining 4- and 6-week data) cohorts. (**P**), Region-specific histopathological scores representing the sum of scores from NP, AF, EP, and interface regions were statistically analyzed using two-way ANOVA with Šidák’s multiple-comparison tests. (**Q**) Cumulative histopathological scores represent the sum of the NP, AF, EP, and interface regions in the IVDs of control and tail-looped cohorts sectioned in the coronal plane. (**R**) Proportion of NP cells with reticular, small, or CLC phenotype in the control and tail-looped cohorts. Statistical significance in (**Q**,**R**) was determined using a mixed-effects model and Šidák’s multiple-comparison tests. Data points represent individual discs. Error bars indicate mean ± SD. Asterisks denote statistical significance. * *p* < 0.05, ** *p* < 0.01, **** *p* < 0.0001. NP, nucleus pulposus. AF, annulus fibrosus. EP, endplate.

H&E staining displayed a reticular-shaped and evenly spread NP, concentric AF lamellae, and intact EPs in the discs of controls (Figure 4A). In looped discs, H&E staining of the coronal sections revealed mild NP narrowing and focal disruption of the inner AF at both 4 and 6 weeks post-surgery (Figure 4B,C). In contrast, the overall disc contours and EP integrity were preserved following the tail-loop immobilization procedure.

Picrosirius Red staining demonstrated red birefringence, indicating strong and organized collagen in the AF lamellae of the controls (Figure 4E) and at 4 weeks post-tail-looping (Figure 4E). At 6 weeks post-tail-looping, an increased green signal indicating reduced collagen quality was observed in the inner AF (Figure 4F), being similar to the tensioned side of the AF in the sagittal plane (Figure 3E,F). The alterations in the area and volume of red-to-green collagen bundles, together with changes in fiber packing and orientation, observed in the tail-looped cohorts in both the sagittal and coronal planes demonstrate the disorganization of collagen fibrils and reduced density following static compressive immobility.

Safranin O–Fast Green staining revealed intense green staining in the AF of controls (Figure 4G), which was progressively reduced from 4 to 6 weeks post-tail-looping (Figure 4H,I). Safranin O staining was homogenous in the NP of controls (Figure 4G) but was heterogeneous with increased peripheral staining and reduced central intensity in the tail-looped NP, indicating uneven proteoglycan distribution following static compression and immobility (Figure 4H,I).

Next, we conducted morphometric analysis to quantify structural changes in the coronal plane following tail-loop immobilization. For these analyses, we combined data from the 4- and 6-week post-looping time points and compared them with those of the sham controls. In line with these matrix changes, quantitative morphometry of the Co9/10 discs showed a significant decrease in NP area in the tail-looped IVDs compared to sham controls, even in the coronal plane (*p* < 0.05, Figure 4J). However, no significant differences were observed in the NP width-to-height aspect ratio and NP cell number following tail-loop immobilization compared to controls (Figure 4K,L). No changes were observed in the AF area, which was comparable between cohorts when analyzed in the coronal plane (Figure 4M). An increase in the AF width-to-height aspect ratio was noted in the looped disc compared to sham controls in the coronal plane (*p* < 0.05, Figure 4N). No differences were observed in AF cell numbers between cohorts when analyzed in the coronal plane (Figure 4O, Supplemental Figure S2A).

Next, we analyzed histopathological changes in the coronal plane across the Co8 to Co10 IVDs. Region-specific histopathological scoring revealed modest and consistent degeneration in looped discs, driven primarily by NP and AF subscores and disruption of the NP-AF interface (*p* < 0.0001). In contrast, EP scores were unchanged (Figure 4P). Cumulative histopathological analysis also revealed mild pathological changes in the looped discs compared with controls (*p* < 0.0001; Figure 4Q).

Considering that no change in the number of NP cells was observed following tail-loop immobilization either in the sagittal or coronal plane, but a modest increase in histopathological changes was noted, next, using the shape of NP cells as a proxy for their healthy state, we quantified changes in NP cells from healthy reticular to mature small-sized and pathological CLC phenotypes [9]. At six weeks following tail-loop immobilization, the results showed early changes in NP phenotype, with a decline in reticular-shaped NP cells (*p* < 0.0012) and an increase in the proportion of small and mature NP cells (*p* < 0.0023; Figure 4R). There was no change in the proportion of CLC-NP cells between cohorts.

These data show that analysis in the coronal plane captures the structural and histological changes in the compressed and immobile discs but underestimates the asymmetric geometric deformation evident in sagittal analyses.

### 3.5. Static Compression and Immobilization Suppress SHH Signaling in NP Cells

**Figure 5:**
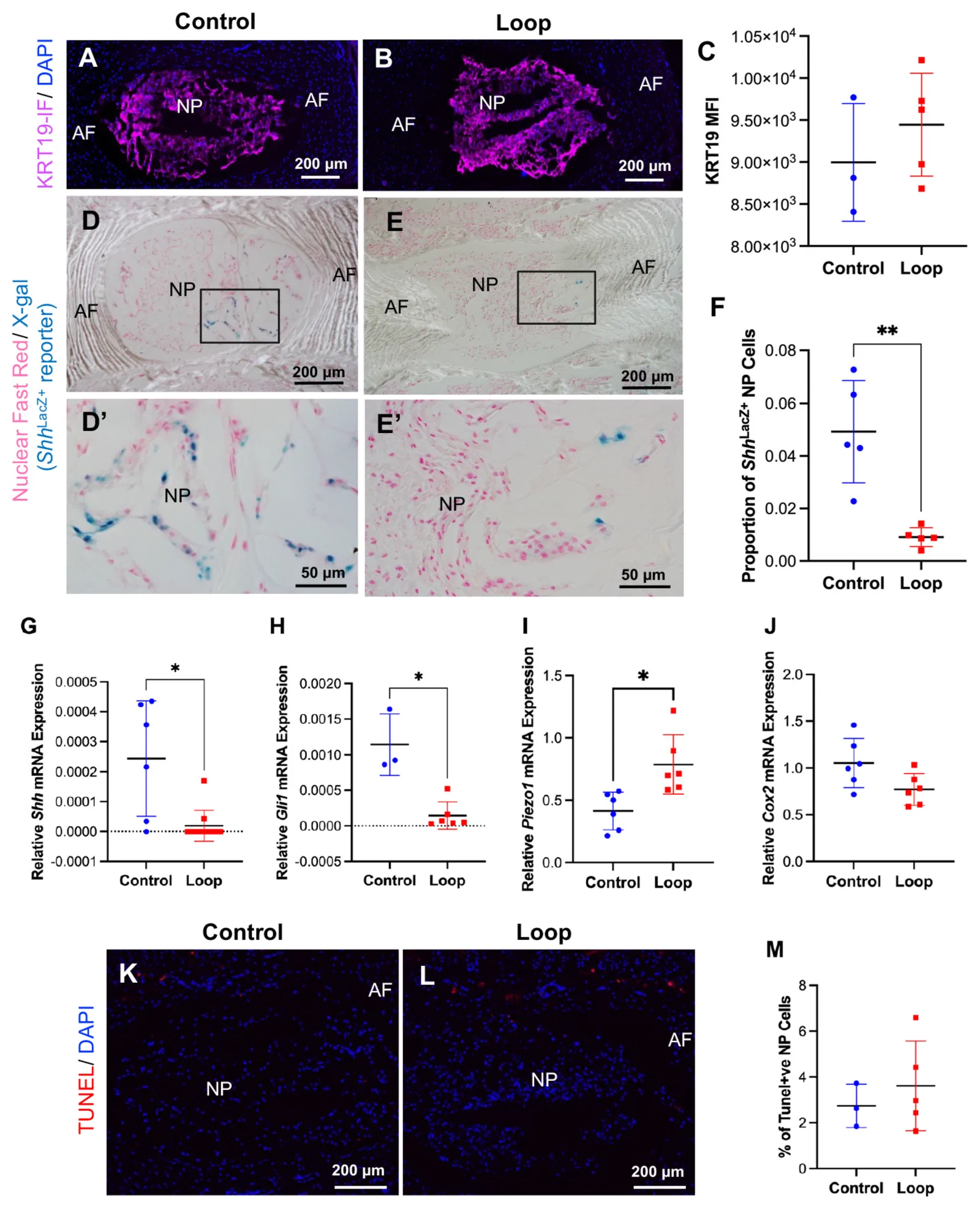
Tail-loop immobilization suppresses SHH signaling in NP cells. (**A**,**B**,**D**,**E**) Representative mid-coronal sections of Co9/10 coccygeal IVDs from sham controls (*n* = 3 to 5) and tail-looped *Shh*-LacZ reporter mice (*n* = 5). (**A**,**B**) KRT19 immunostaining (purple) with DAPI (blue) as nuclear counterstaining. (**C**) Quantification of the KRT19 mean fluorescence intensity (MFI) in NP cells. (**D**,**D’**,**E**,**E’**) X-gal-stained (LacZ+ blue nuclei) with nuclear Fast Red counterstaining. (**D**,**E**) captured using a bright-field camera and differential interference contrast to visualize disc structure. (**D’**,**E’**) are 40× magnification images captured of boxed regions shown in (**D**,**E**), respectively. (**F**) Quantification of the proportion of *Shh*-expressing (LacZ+) NP cells in control and looped discs. (**G**–**J**) Multiplex qPCR analysis of NP cells from control (*n* = 3 to 6) and tail-looped (*n* = 6) coccygeal IVDs for *Shh* (**G**), *Gli1* (**H**), *Piezo1* (**I**), and *Cox2* (**J**) mRNA expression relative to the housekeeping gene *Gapdh*. (**K**,**L**) Representative mid-coronal sections of Co9/10 coccygeal IVDs from sham controls (**K**) and tail-looped (**L**) mice stained for TUNEL (red) and nuclei counterstained with DAPI (blue). (**M**) Quantification of the proportion of TUNEL+ in NP cells. Statistical analysis was performed using an unpaired Welch’s *t*-test. Data points represent individual discs. Error bars indicate mean ± SD. Asterisks denote statistical significance. * *p* < 0.05, ** *p* < 0.01. NP, nucleus pulposus. AF, annulus fibrosus.

The effect of static compressive loading on NP homeostasis was assessed by determining the expression of cytokeratin 19 (KRT19/CK19), a well-established marker of NP cells that is consistently expressed throughout terminal differentiation [9]. Immunostaining demonstrated similar KRT19 immunoreactivity in NP cells from both the tail-looped and sham-operated cohorts (Figure 5A–C).

Next, we assayed SHH expression in NP cells using X-gal staining and *Shh*-LacZ reporter mice at the Co8/9 levels, which were structurally most impacted. Coronal sections in sham controls exhibited several LacZ+ NP cells, identifying the population of *Shh*-expressing cells (Figure 5D,D’). This is consistent with our previous observation that the subset of *Shh*-expressing NP cells declines with postnatal age [9]. Tail-looped discs showed a markedly reduced LacZ+ subset of *Shh*-expressing NP cells following immobilization (Figure 5E,E’). The quantification of LacZ+ NP cells relative to the total number of NP cells in the Co8/9 IVDs revealed a significant reduction in the proportion of *Shh*-expressing NP cells following tail-loop-mediated static compression (*p* < 0.01; Figure 5F).

To test whether the decline in *Shh* expression was due to geometric alterations in disc mechanics or to overall compression and immobility, NP cells were isolated from all IVDs within the looped regions, including levels that were compressed and immobile but modestly affected at the structural level. The qPCR results corroborated a parallel decrease in *Shh* mRNA expression in the NP cells of all looped discs compared to controls (*p* < 0.05; Figure 5G). Furthermore, multiplex qPCR analysis revealed decreased *Gli1* expression, a known hedgehog signaling target [13,42,43,44], in NP cells, demonstrating a decline in *Shh* signaling following static compression and immobilization in the looped discs compared to controls (*p* < 0.05; Figure 5H).

Given that tail-looping alters disc mechanics, the expression of the mechanosensitive marker *Piezo1* in NP cells was subsequently analyzed. Aberrant *Piezo1* expression is associated with IVD degeneration, while its loss has been shown to prevent disc degeneration [45,46]. The quantitative PCR (qPCR) results demonstrated that normal NP cells from control discs expressed *Piezo1*. In contrast, *Piezo1* expression was significantly increased in NP cells subjected to tail-loop immobilization-mediated constant compression (*p* < 0.05; Figure 5I). This upregulation of Piezo1 expression correlated with decreased *Shh* and *Gli1* expression, suggesting a potential association between mechanosensing and hedgehog signaling pathways in regulating IVD homeostasis.

Mechanical stress or injury can induce inflammation in IVD cells; therefore, the expression of Cyclooxygenase-2 (Cox2) was assessed in NP cells. Elevated levels of inflammatory cytokines and growth factors following injury or pathology are known to increase *Cox2* expression, particularly in degenerated IVD cells. The qPCR analysis revealed no significant changes in *Cox2* mRNA levels in NP cells between cohorts. These findings suggest that early time points following tail-loop immobilization may induce cellular and molecular changes in NP cells but do not result in inflammation (Figure 5J).

Given that tail-looping-mediated IVD compression resulted in reduced SHH expression, a TUNEL assay was performed to assess NP cell viability. The TUNEL assay showed no significant effect on NP cell viability at 6 weeks after tail-looping compared with sham controls (Figure 5K–M). These findings indicate that the earliest event following compressive immobility is a reduction in Shh expression by NP cells, which may subsequently contribute to the degenerative changes observed at the later time points in previous studies [27,39].

Overall, these results indicate that sustained mechanical immobilization first suppresses Shh signaling at both the cellular and transcriptional levels and increases the expression of the *Piezo1* mechanosensing marker, implicating mechanical stress and immobility as regulators of NP homeostasis via the SHH signaling pathway.

### 3.6. Tail-Loop Immobilization Induces a Focal Annular Tear Phenotype

**Figure 6:**
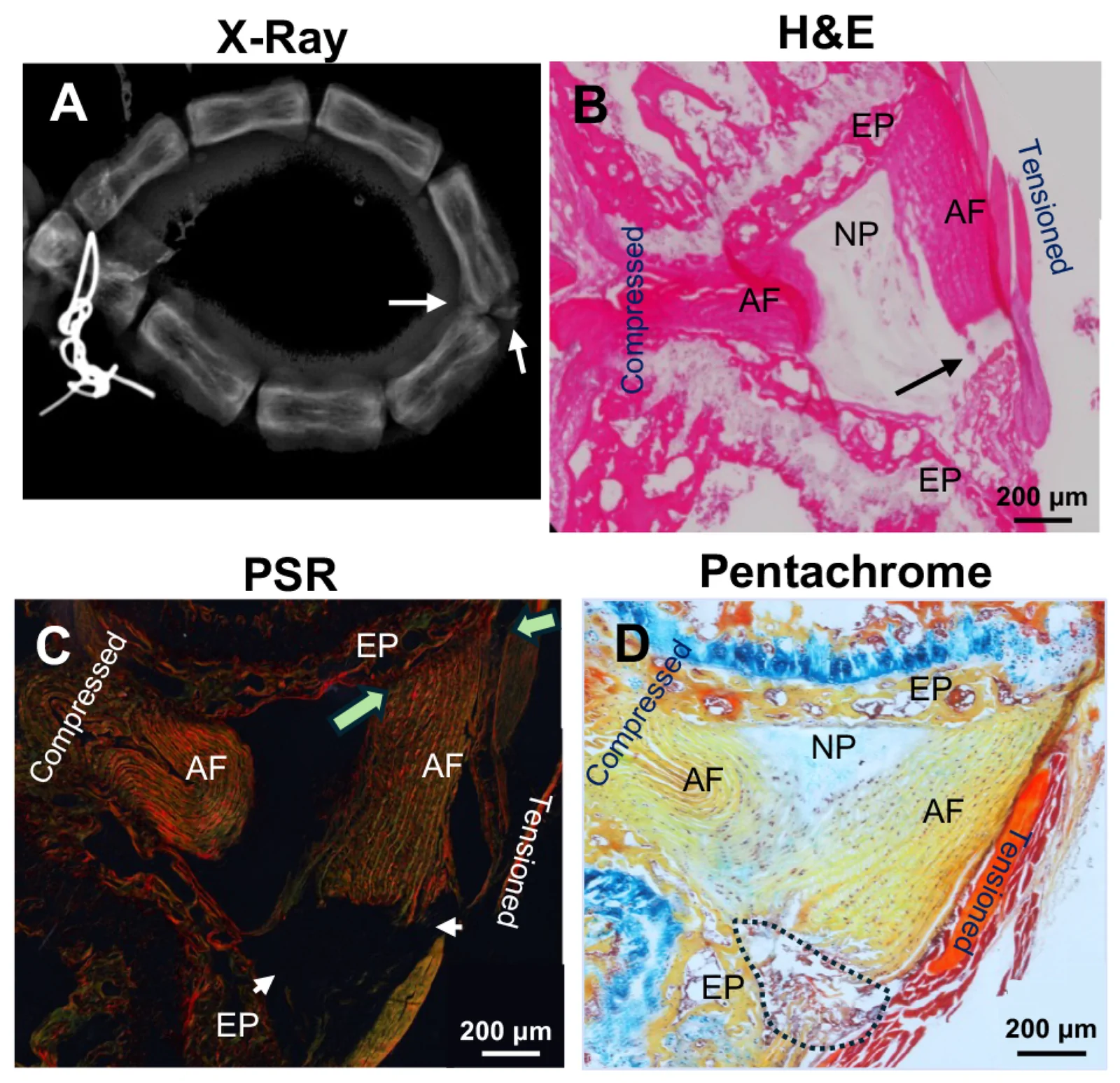
Tail-loop immobilization induces a focal annular tear phenotype. Representative X-ray image of a looped segment (*n* = 2) showing an endplate fracture (white arrow, (**A**)). Representative images of sections prepared in the sagittal plane of tail-looped coccygeal IVDs stained with H&E (**B**), Picrosirius Red (PSR, (**C**)), and Movat’s Pentachrome (**D**), showing a focal annular tear extending from the outer tensioned AF toward the NP region. Black arrow (**B**) indicates AF tears and NP cell loss; white arrowheads (**C**) mark collagen fiber detachment along the tear plane, with the green arrow showing a normal fiber trajectory into the EP; black dotted line (**D**) outlines neovascularization at the AF-EP boundary, where a tear had occurred. NP, nucleus pulposus. AF, annulus fibrosus. EP, endplate.

In a subset of tail-looped IVDs (*n* = 2, 11.11%), we identified discrete endplate fracture and annular tear phenotypes visible in the sagittal plane that were not observed in sham controls (Figure 6). An X-ray image of the looped region shows the dissociation of the EP region from one side of the disc that is mid-way in the loop (white arrow, Figure 6A). H&E staining revealed focal fissures extending from the inner AF of the tensioned side toward the NP region, with localized IVD architectural disruption along with NP cell loss (black arrow, Figure 6B). Picrosirius Red staining demonstrated the detachment and reorientation of collagen fibers (white arrow heads) along the tear plane, which would normally extend from the AF and integrate in the EP region (green thick arrow, Figure 6C). Movat’s Pentachrome staining confirmed the loss of NP cells and annular tears along the AF-EP boundary, along with neovascularization in the torn region (black dotted line, Figure 6D). Such annular tears in the tail-loop model have previously been reported using the suture model [39]. These findings indicate that chronic asymmetric loading can precipitate localized AF rupture, potentially leading to NP cell loss or to the infiltration of non-resident cells into the NP space. The migration of non-resident cells into the NP space could impact IVD pathophysiology that is independent of static compression or immobility. The IVDs with annular fissures were excluded from this study.

## 4. Discussion

We have identified static mechanical compression as a direct regulator of SHH expression in the postnatal NP and demonstrate that sustained immobilization is sufficient to initiate early disc degeneration. Using a *Shh*-LacZ reporter mouse allele combined with a multi-level tail-loop paradigm, we show that chronic mechanical constraint induces reproducible disc wedging, the region-specific remodeling of the NP and AF compartments, and collagen disorganization. Importantly, these structural changes are accompanied by a marked reduction in SHH-expressing NP cells and decreased *Shh* and *Gli1* transcription, establishing the mechanical repression of a developmental signaling pathway as an early event in disc degeneration.

SHH is a central regulator of IVD development [47] and postnatal growth and maintenance, where its continued expression by notochord-derived NP cells supports cell proliferation and matrix homeostasis and coordinates signaling with the surrounding AF and EP tissues [12,13,14,15]. While a decline in SHH expression is associated with disc aging and degeneration [4,12,15], the upstream signals governing this decline remain unclear. Our data demonstrate that mechanical immobilization is sufficient to suppress SHH expression in vivo, positioning SHH as a mechanosensitive signaling node in the adult disc, rather than a passive marker of degeneration. This is supported by the qPCR analysis of pooled NP cells from all discs, including those with no quantifiable geometrical changes, indicating that static compression and immobility that naturally occur in the lumbosacral and sacral discs are the main drivers of the suppression of *Shh* and *Gli1* expression by NP cells.

Importantly, the degeneration induced by tail-looping within 4 to 8 weeks analyzed in this study is mild but reproducible and captures a window of early disease that precedes overt cell loss or EP failure, and it is consistent with previous reports [26,27,39]. Notably, a decline in SHH signaling occurred in the absence of significant NP cell death, indicating that mechanical cues primarily alter the NP cell state at the molecular level, rather than viability at the early stages of degeneration. These findings are consistent with prior observations that NP cells shift toward a chondrocyte-like phenotype as SHH expression declines and are more prevalent in the lumbosacral discs [12,13,14,15], suggesting that static compression accelerates this process by directly repressing SHH output. Furthermore, marked reductions in NP area and the width/height aspect ratio were observed, indicating that geometric deformation and altered matrix organization—not the number of NP cells—are the primary early consequences of static loading. This temporal separation suggests that mechanical dysregulation initiates degeneration before traditional hallmarks of cell loss become evident. However, prolonged immobility and compression would likely lead to a decline in the number of NP cells, as noted in previous studies [27,39].

Our findings reinforce and extend prior studies using the tail-loop model [26,27,39]. The tail-loop model originally showed that asymmetric caudal loading induces progressive structural degeneration in murine discs analyzed in the sagittal plane [27]. The tail-loop model demonstrated that AF weakening is a critical contributor to disc instability under similar loading conditions. Consistent with these reports, our analyses in the sagittal plane reveal pronounced asymmetry between compressed and tensioned AF regions. This feature is underestimated in analyses conducted in the coronal plane. The compressed side of the AF showed altered width-to-height ratios and collagen disorganization. At the same time, the tensioned side of the AF displayed shifts toward thinner, weaker collagen fibrils, as evidenced by increased green birefringence under polarized light. In addition, gradual deformity of the disc structure and geometry was observed toward the center of the loop. Moreover, although more subtle, structural changes were evident in the coronal plane, disc wedging was not. These findings indicate that distinct mechanical environments within a single disc elicit divergent matrix responses, highlighting the importance of accounting for spatial heterogeneity when assessing disc degeneration using the tail-loop model. Furthermore, the current study demonstrates that the effects of static loading and immobility extend beyond the AF and suppress SHH signaling at the molecular level—a pathway that is fundamental to NP cell homeostasis and matrix maintenance across all components of the IVD [9,12,13,15].

Epidemiological and pre-clinical studies consistently show that lumbosacral IVDs degenerate earlier and more severely than adjacent lumbar levels [3,4,5,6,7,8,9,48]. However, the biological basis for this regional susceptibility has remained unresolved. Our findings provide a mechanistic framework linking restricted mobility and sustained compression—hallmarks of the lumbosacral environment—to accelerated NP dysfunction. Tail-loop immobilization creates a mechanically constrained segment analogous to the lumbosacral junction, characterized by an increased compressive load, reduced excursion, and spatially asymmetric stress distribution. Considering that humans are bipedal, whereas rodents and other animals used in pre-clinical models of disc degeneration are quadrupedal, our findings that static compression and a lack of mobility drive disc degeneration by suppressing SHH signaling explain why the lumbosacral region is consistently the most susceptible to early pathologies across all models [3,4,5,6,7,8,9].

Based on our findings, we propose a feed-forward mechanobiological model in which chronic static compression suppresses SHH expression in NP cells, leading to impaired matrix maintenance and reduced signaling support to surrounding AF and EP tissues in the lumbosacral and sacral discs. These changes, in turn, exacerbate abnormal load transmission, further reinforcing SHH repression and promoting progressive degeneration. In this model, SHH signaling functions as a molecular integrator of mechanical history, translating sustained physical constraint into long-term biological consequences. This framework helps to reconcile why sacral discs—subjected to maximal immobilization—completely lose SHH expression and undergo fusion by skeletal maturity. In contrast, lumbosacral discs exhibit intermediate phenotypes characterized by early degeneration. It also provides a conceptual basis for why interventions that restore motion or modulate load distribution may have disease-modifying potential if applied early, before the irreversible loss of the NP molecular signature occurs. This is supported by previous reports showing that dynamic pressurization was beneficial for SHH expression in bovine disc NP cells ex vivo [49].

PIEZO1 and its downstream targets YAP/TAZ are known markers of mechanosensing in the disc [45]. Aberrant expression of *Piezo1* is related to disc degeneration [46]. By demonstrating that the mechanical repression of SHH signaling was associated with increased expression of mechanosensing marker *Piezo1*, known to be associated with IVD degeneration, this study provides a mechanistic link between chronic compression and disc degeneration. Moreover, there were no changes in ECM content or cell survival rates, indicating a direct effect of mechanical suppression on SHH expression. Hence, this study shifts the paradigm from viewing disc degeneration solely as wear and tear to understanding it as a failure of mechanosensitive developmental signaling in adulthood. These findings emphasize the importance of considering regional biomechanics in both experimental design and therapeutic development.

Future studies will be required to define the upstream mechanotransduction pathways that couple static loading to SHH repression, including potential roles for cytoskeletal tension, chromatin remodeling, and mechanoresponsive transcriptional regulators. Additionally, it will be interesting to determine whether static compression alone or immobility alone is sufficient to repress SHH expression by NP cells. Additionally, determining whether the restoration of SHH signaling can rescue mechanically compromised discs will be critical for evaluating its potential as an early therapeutic target. Moreover, the observation of a focal annular tear phenotype in a subset of looped discs reveals a limitation of the tail-loop model. AF tears or EP fractures would lead to the infiltration of surrounding or immune cells into the disc space, which could interfere with the analysis of effects due to compression and immobility on these discs. Hence, when using the tail-loop model, discs with AF tears or EP fractures should be excluded from the study.

## 5. Conclusions

In summary, our work establishes the role of mobility and mechanosensing as regulators of SHH expression by NP cells and that static mechanical immobilization directly suppresses SHH expression and signaling in NP cells. The magnitude and consistency of SHH suppression at mechanically constrained levels parallel prior observations that lumbosacral and sacral discs lose SHH earlier than more mobile lumbar levels. Together, these findings support a model in which restricted mobility and sustained compression accelerate disc aging by prematurely silencing developmental signaling programs like SHH required for NP homeostasis. By linking biomechanics to developmental signaling, this study offers new insight into how disc degeneration is initiated and identifies new avenues for early intervention.

## Data Availability

No new datasets were generated. Data are contained within the article or Supplementary Materials.

## Supplementary Materials

The following supporting information can be downloaded at https://www.mdpi.com/article/10.3390/cells15171506/s1. Supplemental Figure S1: Representative H&E-stained sagittal sections of coccygeal spine segments from control (A) and tail-looped (B) cohorts; Supplemental Figure S2: Representative fluorescence microscopic images of coccygeal IVDs sectioned in the sagittal plane (A) and coronal plane (B) of sham controls (left column) and tail-looped (right column) cohorts. Sections were stained with DAPI and imaged using epifluorescence and DIC filters. NP, nucleus pulposus. AF, annulus fibrosus.
