# Supplementary material for: Mechanical Suppression of Sonic Hedgehog Signaling by Nucleus Pulposus Cells Underlies Early Disc Degeneration in Mouse
Source: Cells. 2026 Aug 22;15(17):1506. doi: 10.3390/cells15171506 (PMC13565538; doi:10.3390/cells15171506)
Supplement: Supplementary file 1 [file cells-15-01506-s001.zip › cells-4328540-supplementary.pdf]

Supplemental Figure S1

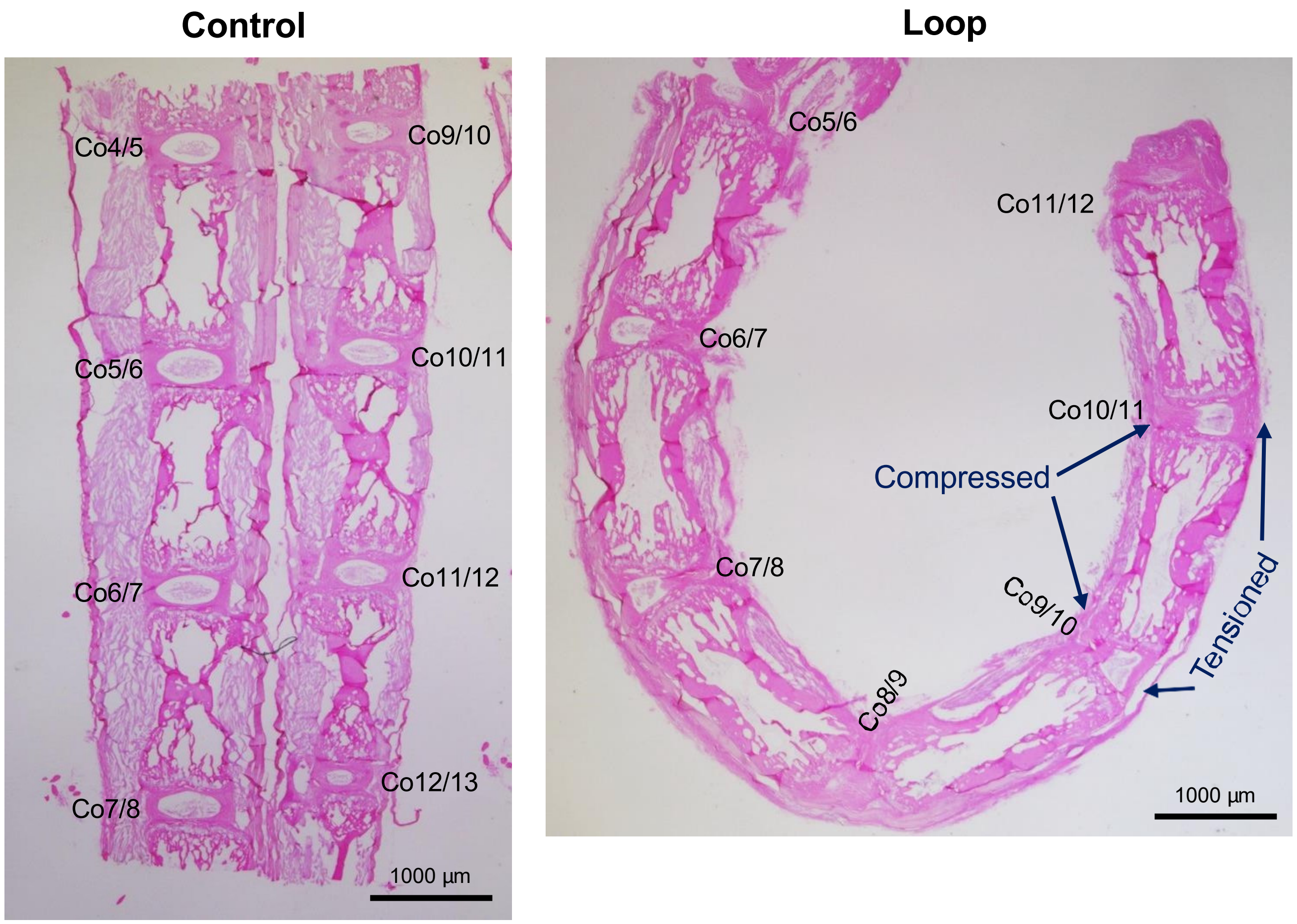

Supplemental Figure S1. Representative H&E-stained sagittal sections of coccygeal spine segments from control (**A**) and tail-looped (**B**) cohorts.

Supplemental Figure S2

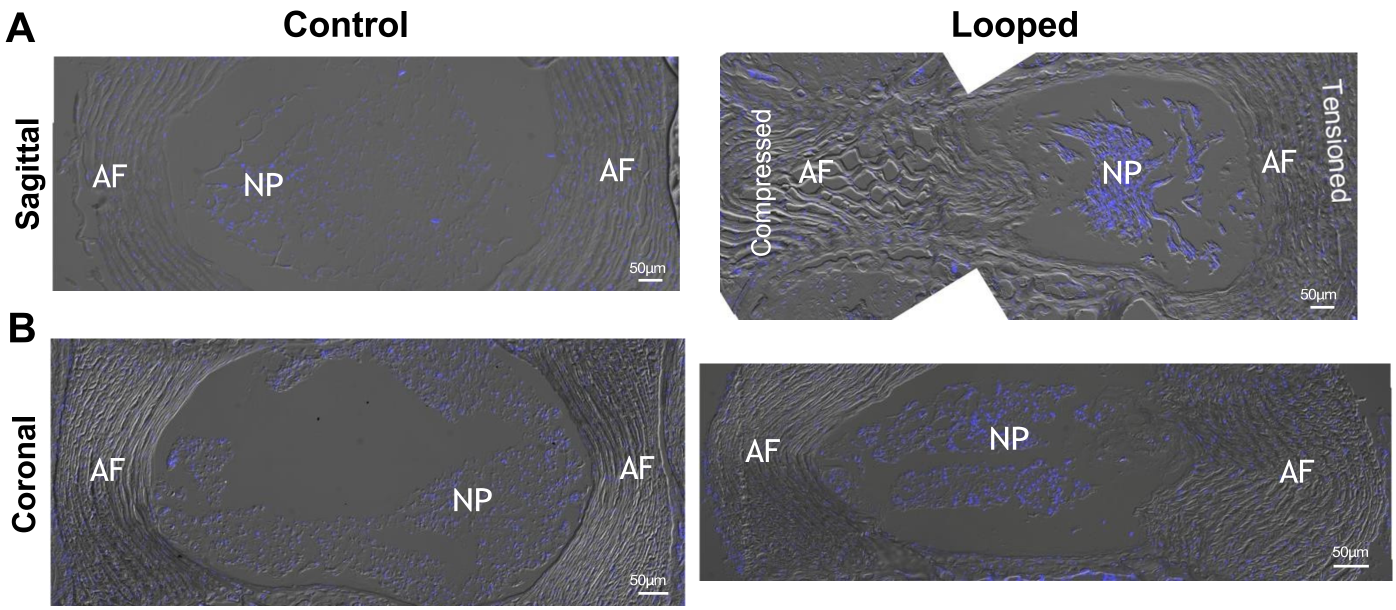

Supplemental Figure S2. Representative fluorescence microscopic images of coccygeal IVDs sectioned in the sagittal plane (**A**) and coronal plane (**B**) of sham controls (left column) and tail-looped (right column) cohorts. Sections were stained with DAPI and imaged using epifluorescence and DIC filters. NP, nucleus pulposus. AF, annulus fibrosus.
